# Supplementary material for: Learning curve and surgical outcome of robotic assisted colorectal surgery with ERAS program
Source: Sci Rep. 2022 Nov 29;12:20566. doi: 10.1038/s41598-022-24665-w (PMC9709162; doi:10.1038/s41598-022-24665-w)
Supplement: Supplementary file 1 — Supplementary Information 1. [file 41598_2022_24665_MOESM1_ESM.zip › LSC Raw data-Ñ[▒K/095/N_ñw╝╨░O▒Kñσ.pdf]

W55 / W55

臺中榮民總醫院
手術室護理記錄

[redacted]

基本資料

- 室別： 二醫手術室-31室，手術科別： CRS，手術類別： 常規，日期： 2020/09/30
- 進入時間： 1120 ； 麻醉開始時間： 1125 ； Time Out時間： 1250 ； 劃刀時間： 1250 ； 手術結束時間： 1925 送出時間： 1930 ；
- 麻醉方式： GE

  診斷：T colon ca.

  手術名稱：Laparoscopic right colectomy and anastomosis
  Laparoscopic cholecystectomy

  手術局部用藥：nil

  術前預防性抗生素：
  - 抗生素名稱一： Veterin 1GM IVD(由麻醉科護理人員給予)
  - 時間： 1210
  - 上次簽章: 吳明柔

  ，追加預防性抗生素：
  - 抗生素名稱一： Veterin 1GM IVD(由麻醉科護理人員給予)
  - 時間： 1640
  - 記錄者： 王毓均
- 手術醫師： 林俊余，楊曙榮，陳瑢夫，林陳彥
- 麻醉醫師： VS 賴慧卿
-
  - 刷手護士A ： 林宜旻，時間： 1120，接替刷手一： 吳雅華，時間： 1500，接替刷手二： 王惟瑩，時間： 1830
  - 流動護士A ： 吳雅華，時間： 1120，接替流動一： 吳明柔，時間： 1230，接替流動二： 王毓均，時間： 1800

皮膚狀況

A-V Shunt： 無

術前： 不完整

部位(術前)：

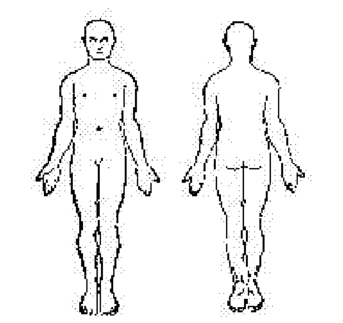

右手肘內側破皮結痂、雙下肢脫皮

記錄者： 上次簽章: 吳雅華

術後： 不完整

部位(術後)：

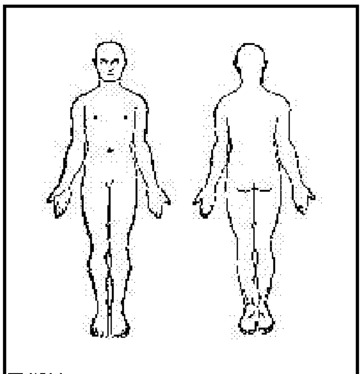

同術前

記錄者： 上次簽章: 王毓均

手術準備

手術臥式 單一手術臥式

-
  - 手術臥式： 仰臥
  - 使用附件： 矽膠枕頭
  - 皮膚準備： 10%Aq Betadine , Hibiscrub , 2% Chlorhexidine
  - 電燒： 使用電燒: 電極片位置： 其它： 迴路電極墊，記錄者： 上次簽章: 吳雅華

止血帶T： 無

植入物

- 植入物名稱/號碼/數量/部位：hyalobarrier/10c.c./壹支/pelvic

, 記錄者： 上次簽章: 王毓均

標本送檢

- 細菌培養 厭氧，採取部位: 1. GB BILE，數量： 1
- 細胞學 無
- 冰凍切片 無
- 病理標本
  組織名稱一：A-T COLON
  ,
  組織名稱二：MIDDLE COLIC ROOT
  ,
  組織名稱三：GB
- 截肢 無

標本交付 標本： 3 袋，標本： 零 盒，標本： 零 瓶，細胞學： 零 管，Culture Tube： 1 管，被交付醫師： R 陳瑢夫，

- 記錄者： 上次簽章: 王毓均
- 時間： 1900

傷口術後敷料

傷口敷料： 傷口敷料： 無

- 請於圖上標示部位：

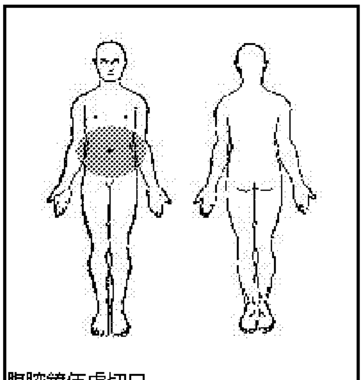

腹腔鏡伍處切口

- 傷口敷料： Mepore

導管

導管	天數	導管資訊	插入日期	插入時間	更換日期	拔除日期	拔除時間	拔除原因	預計拔除/更換	預計拔除/更換日期
CVC(1)	15	深度:外露7.3cm; 部位:頸部; 象限:LU;	2020/09/16	1145						
A-line (1)	1	號碼:20 G; 部位:前臂;	2020/09/30	1130						
Sump NG Tube(1)	1	號碼:16 Fr.; 部位:胃; 註記:FIX 58CM;	2020/09/30	1125						
Penrose Drain(1)	1	號碼:12 mm.; 部位:肝下; 註記:右腹部;	2020/09/30	1830						
Foley(1)	1	號碼:16 Fr.; 部位:膀胱;	2020/09/30	1120						

用物計數

- 手術中出血量： 350 c.c.
- 手術中尿量： 1000 c.c.
-
  - 其它： 無

用物計數： 多切口同時縫合

- 1.器械： 正確，2.縫針： 正確，3.刀片： 正確，4.紗布： 正確，5.腹部墊： 未使用，6.紗球： 未使用，7.PEANUTS ： 未使用，8.紗條： 未使用，9.絲帶： 未使用，10.針頭： 未使用，11.Vessel loop： 未使用，12.Boots： 未使用，13.Jelco： 未使用，14.棉條： 未使用，
  - 第一次：
    - 刷手護士 ： 吳雅華
    - 流動護士： 上次簽章: 王毓均
    - 時間： 1810

  ,
  - 第二次：
    - 刷手護士 ： 吳雅華
    - 流動護士： 上次簽章: 王毓均
    - 時間： 1830

  ,
  - 第三次：
    - 刷手護士 ： 王惟瑩
    - 流動護士： 上次簽章: 王毓均
    - 時間： 1900

備註

備註： NIL

- 記錄者： 上次簽章: 吳明柔
- 時間： 1330

填寫護理人員 王毓均

正式摘要

W55 / W55

doctorRank:B
